# Supplementary material for: Patterns of Intron Gain and Loss in Fungi
Source: PLoS Biol. 2004 Nov 30;2(12):e422. doi: 10.1371/journal.pbio.0020422 (PMC532390; doi:10.1371/journal.pbio.0020422)
Supplement: Table S1 — Also available at http://genes.mit.edu/NielsenEtAl/. (4.3 MB ZIP). [file pbio.0020422.st001.zip › NielsenEtAl/html/1304.html]

AN1284.1.NCU02044.1.MG10957.1.FG02764.1


```
 CLUSTAL W (1.82) Multiple Sequence Alignments - Introns Inserted


Sequence 1: NCU02044.1	403 aa
Sequence 2: MG10957.1	408 aa
Sequence 3: FG02764.1	394 aa
Sequence 4: AN1284.1	580 aa
Alignment Length: 595 aa
Number Identitical Residues: 282 aa
Alignment Score (without introns) 12113


MG10957.1 	MPPKKAKEEEKILLGRPGNNLKSGI0VGLANVGKSTLFQAITKCNLGNPA~VRHQPRMQP
NCU02044.1	MPPKKQVVEEKILLGRPGNNLKSGI0VGLANVGKSTLFQAITKCNLGNPA0LTHPPARQ-
FG02764.1 	MPPKKQVVEEKIPLGRPGNNLKSGI0VGLANVGKSTLFQAITKSNLGNPA0---------
AN1284.1  	MPPKKAPVQEKVLLGRPGNNLKSGI0VGLANVGKSTLFQAITKSGLGNPA~---------
          	*****   :**: ************ *****************..*****          

MG10957.1 	RLAFC0NFPYATIDPEEARVIVPDDRYDWLCQKYNPKSRVPANLTVYDIAGLTRGASTGA
NCU02044.1	-----~NFPYATIDPEEARVIVPDERFDWLCEKYQPKSRVPANLTVYDIAGLTRGASTGA
FG02764.1 	-----~NFPYATIDPEEARVVVPDDRFDWLVEKYKPKSVVPANLTVYDIAGLTRGSSTGA
AN1284.1  	-----~NFPYATIDPEEAKVIVPDERFDWLCEHYKPKSKVPANLTIYDIAGLTRGASTGA
          	      ************:*:***:*:*** ::*:*** ******:*********:****

MG10957.1 	GLGNAFLSHIRAVDAIFQVVRCFDDAEIIHVEGDVNPTRDLDIISEELRLKDIEFVEKAL
NCU02044.1	GLGNAFLSHIRAVDAIFQVVRAFDDAEIIHVEGDVNPTRDLDIISEELRLKDIEFVEKAL
FG02764.1 	GLGNSFLSHIRAVDAIFQVVRCFDDAEIIHIEGDVNPTRDLDIISEELRLKDIEFTEKAL
AN1284.1  	GLGNAFLSHIRAVDAIFQVVRCFDDAEIIHVEGDVDPVRDLSIISEELRIKDIEFVEKAL
          	****:****************.********:****:*.***.*******:*****.****

MG10957.1 	ENQKKKTRQGGQSLEQKKAREEEATIEKILAWLKDGKDVRKGNWGPKE0VEVINPLFLLT
NCU02044.1	ENQKKKTRQGGQSLQMKQWKEEEATIEKILAHLKDGKEVRKGTWGPKE0IEVINPLFLLT
FG02764.1 	ENQKKKTRMGGQSLEQKKAVQDQATLEKVLAWLNDGKDVRKGNWGPKE0IEVINPLFLLT
AN1284.1  	ENLKKQTRRGGQSLEMKKLREDEATTAKVLEFLQAGKNIRHGDWTPKE0VEVINPLFLLT
          	** **:** *****: *:  :::**  *:*  *: **::*:* * *** :**********

MG10957.1 	AKPVVYLVNLSEKDYIRKKNKHLPKIAEWIKEHATGDPIIPISVSFEERLTRFETEAEAL
NCU02044.1	AKPVVYLVNLSERDYIRKKNKHLPAIAAWINEHAKGDPIIPLSVSFEERLTRFETEAEAA
FG02764.1 	AKPVVYLVNLSEKDFIRKKNKHLPKIVEWINENAKGDPLIPISVSYESRLTLCETEAESK
AN1284.1  	AKPVVYLVNLSEKDYIRQKNKYIPKLMEWIKTNSPGDSILPISASFEERLTQFETEAEAE
          	************:*:**:***::* :  **: :: **.::*:*.*:*.***  *****: 

MG10957.1 	EECKNVGAESALPKVIIQMRKALNLGSFFTTGTDEVRQWTIRNGTKAPQAAGVIHGDFEK
NCU02044.1	EECKKVGAESALPKIIIQMRKALNLGSFFTTGPDEVRQWTIRNGTKAPQAAGVIHTDFEK
FG02764.1 	EEQKTAGADSVLPKVILQMRKTLQLGSFFTVGPDEVRQWTIRNGTKAPQAAGVIHNDFEK
AN1284.1  	EECKRLGTKSGLPKAIVQMRQVLNLASFFTTGADEVRQWTIRKNTKAPAAAGVIHGDFEK
          	** *  *:.* *** *:***:.*:*.****.*.*********:.**** ****** ****

MG10957.1 	TFIQCIVYNYNVLKELG-DEAEVKAKGKVMTKGKDYVVEDGDILLIKAGAAKG-~-----
NCU02044.1	TFIQAVVYNYNVLRELGGDEAEVKAKGKVMTKGKDYVVEDGDIILFKAGAAKS-~-----
FG02764.1 	TFIQALVFNYNILKELG-DESEVKAKGKIMTKGKEYVMEDGDIVHFKAGAAKG-~-----
AN1284.1  	TFIQAIVYNYNTLREYG-DEAAVKAAGKVMTKGKDYIVEDGDILLIKAGAARSY2LVRLL
          	****.:*:*** *:* * **: *** **:*****:*::*****: :*****:.       

MG10957.1 	~---------------------------------------------------~-------
NCU02044.1	~---------------------------------------------------~-------
FG02764.1 	~---------------------------------------------------~-------
AN1284.1  	0ALDSIPPSNRSKVKSSVQRALRQKLVEAHPRCEPYIDEILPKKAQLDAVKL2PDRVTLY
          	 : .: ..:. :. .:: . :  ..  .: . ...  ..  ...:. .: .  ..  :  

MG10957.1 	------------------------------------------------------------
NCU02044.1	------------------------------------------------------------
FG02764.1 	------------------------------------------------------------
AN1284.1  	TIDSTPLFYQPLDGPPIPHLKLLHQFPDMLPTIQIDRGAIRFVLSGAALMAPGLTSPGGR
          	: .::.   .. .... .  .   . ..  .: . . .:     :.::  :.. ::... 

MG10957.1 	------------------------------------------------------------
NCU02044.1	------------------------------------------------------------
FG02764.1 	------------------------------------------------------------
AN1284.1  	LPDAEHALEQGTVVAVKAEGKEEICMVGTLKVGTEEIKAKGKGVAIDDGHYLGDGLWKMH
          	 ..:. : ...:  : .:..... .  .: . .:.. .:.... : ...   ...  .  

MG10957.1 	--
NCU02044.1	--
FG02764.1 	--
AN1284.1  	LD
          	 .
```
